# Three-Dimensional Quantitative Tumor Response and Survival Analysis of Hepatocellular Carcinoma Patients Who Failed Initial Transarterial Chemoembolization: Repeat or Switch Treatment?

**DOI:** 10.3390/cancers14153615

**Published:** 2022-07-25

**Authors:** Yan Zhao, Reham R. Haroun, Sonia Sahu, Ruediger E. Schernthaner, Susanne Smolka, Ming-De Lin, Kelvin K. Hong, Christos Georgiades, Rafael Duran

**Affiliations:** 1 Department of Gastroenterology, First Affiliated Hospital of Xi’an Jiaotong University, Xi’an 710061, China; yanzhao211@163.com; 2 Russell H. Morgan Department of Radiology and Radiological Science, Division of Vascular and Interventional Radiology, The Johns Hopkins Hospital, Sheikh Zayed Tower, Ste 7203, 1800 Orleans St, Baltimore, MD 21287, USA; rehamharoun1989@gmail.com (R.R.H.); sonia.p.sahu@gmail.com (S.S.); ruediger@schernthaner.eu (R.E.S.); khong1@jhmi.edu (K.K.H.); cgeorgi@jhmi.edu (C.G.); 3 Department of Radiology, University of Michigan, 1500 E Medical Center Dr, Ann Arbor, MI 48109, USA; 4 Department of Radiology and Biomedical Imaging, Yale University School of Medicine, 330 Cedar Street, TE 2-230, New Haven, CT 06520, USA; susanne.smolka@charite.de (S.S.); mingde.lin@yale.edu (M.-D.L.); 5 Department of Radiology and Interventional Radiology, Lausanne University Hospital, University of Lausanne, Rue du Bugnon 46, CH-1011 Lausanne, Switzerland

**Keywords:** TACE, transarterial chemoembolization, survival, hepatocellular carcinoma, tumor response, qEASL

## Abstract

**Simple Summary:**

Transarterial chemoembolization is the main therapy for patients with intermediate-stage hepatocellular carcinoma; it has demonstrated efficacy and survival benefits. However, treatment success cannot always be achieved after one treatment session. Using a quantitative 3D tumor-response assessment, we showed that a second transarterial chemoembolization in patients who initially do not respond to therapy results in both objective tumor response and survival benefits. Thus, at least two sessions should be performed before TACE is abandoned and alternative treatments are considered.

**Abstract:**

Objectives: The purpose of this study was to assess treatment responses and evaluate survival outcomes between responders and non-responders after each transarterial chemoembolization (TACE) session using the 3D quantitative criteria of the European Association for the Study of the Liver (qEASL) in hepatocellular carcinoma (HCC) patients. Methods: A total of 94 consecutive patients who underwent MR imaging before and after TACE were retrospectively included. Volumetric tumor enhancement (qEASL) was expressed in cubic centimeters (cm^3^). The Kaplan–Meier method with the log-rank test was used to calculate the overall survival (OS) for the non-/responders. Results: In total, 28 (29.8%) patients showed a response after the first TACE. These responders demonstrated a clear trend toward longer OS compared with the non-responders (36.7 vs. 21.5 months, *p* = 0.071). Of the 43 initial non-responders who underwent a second TACE within 3 months and had complete follow-up imaging, 15/43 (34.9%) achieved a response, and their median OS was significantly longer than that of the 28 non-responders to the second TACE (47.8 vs. 13.6 months, *p* = 0.01). Furthermore, there was no significant difference in OS between the 28 patients who achieved a response after the first TACE and the 15 initial non-responders who achieved a response after the second TACE (36.7 vs. 47.8 months, *p* = 0.701). The difference in OS between the responders and non-responders after the third TACE was not significant (11.4 months vs. 13.5 months, *p* = 0.986). Conclusion: Our study quantitatively demonstrated that a second TACE can be beneficial in terms of tumor response and survival for HCC patients who do not initially respond to TACE.

## 1. Introduction

Hepatocellular carcinoma (HCC) is a major concern and the fourth leading cause of cancer-related deaths [1]. Although surveillance for HCC is performed, most patients are diagnosed at intermediate or advanced stages and curative treatments are limited. Transarterial chemoembolization (TACE) is the most widely used treatment for unresectable HCC and the recommended first-line therapy for intermediate-stage patients in the Barcelona Clinic Liver Cancer (BCLC) staging classification [2]. However, not all intermediate-stage HCC patients achieve similar survival benefits or responses after TACE. There are no definite data about the policy for retreatment in non-responders to TACE. Moreover, although TACE may achieve increased tumor response after repeated therapy, the ideal number of TACE is not known, and therapy is currently discouraged if patients fail to respond to two consecutive TACE sessions [3,4]. 

A seminal study demonstrated that at least two TACE procedures should be performed as improved survival outcomes were obtained with additional treatment, even in patients who did not show a response after the first TACE; the responses were assessed by the modified Response Evaluation Criteria in Solid Tumors (mRECIST) and European Association for the Study of the Liver (EASL) [5]. Recently, using mRECIST, these results were confirmed in a large cohort of HCC patients in which three TACE treatments proved beneficial in obtaining responses from non-responders [6]. 

However, the mRECIST and EASL criteria assume that tumors grow or shrink symmetrically in one or two dimensions. Indeed, most liver tumors undergo heterogeneous growth or necrosis, which limits the applicability of mRECIST or EASL [7]. Quantitative EASL (qEASL) is a new three-dimensional (3D)-imaging biomarker of tumor response, which allows volumetric measurements of the whole-tumor and the enhancing-tumor parts. The superiority of this quantitative imaging biomarker for the assessment of treatment response over current one- or two-dimensional imaging biomarkers, such as RECIST, mRECIST and EASL, has been shown in previous studies [8,9,10,11].

The purpose of our study was to investigate the tumor responses of intermediate-stage HCC patients treated with repeated TACEs, as determined with the 3D qEASL criteria, and compare the overall survival (OS) of the responders and non-responders.

## 2. Materials and Methods

This was a single-institution, retrospective, Institutional Review Board-approved and Health Insurance Portability and Accountability Act-compliant study. Informed consent was waived.

### 2.1. Study Design

Our database (prospectively collected) of HCC patients was reviewed and 135 consecutive patients with intermediate-stage BCLC (stage B) who underwent TACE were included. Patients with concurrent chemotherapy were excluded. The diagnosis of HCC was based on histologic confirmation or clinical-radiological results (typical wash-in/wash-out on dynamic liver imaging) [4]. Patients with compromised performance status (Eastern Cooperative Oncology Group (ECOG) ≥ 1), tumor invasion of the portal vein, or extrahepatic disease were excluded. Patients with poor MR image quality (*n* = 6), infiltrative HCC (*n* = 10), no baseline imaging (*n* = 21), or MR scans using hepatobiliary-specific gadolinium agents (*n* = 4) were excluded. The final study population included 94 patients. 

### 2.2. Transarterial Chemoembolization

Treatment decisions were always discussed on multidisciplinary tumor boards and TACE was considered the best treatment. Experienced interventional radiologists carried out the TACEs. In all cases, hepatic tumor-feeding vessels were selectively/superselectively catheterized using a microcatheter (Renegade 2.4Fr, Boston Scientific, Natick, MA, USA). For conventional TACE (cTACE), Lipiodol (Guerbet, Paris, France) was mixed with doxorubicin (50 mg) and mitomycin C (10 mg) in order to obtain an emulsion (ratio of the mixture: 1:1). Administration of the emulsion was followed by bland embolization (Embosphere; Merit Medical, South Jordan, UT, USA) in order to obtain a substantial reduction in the arteria inflow. For drug-eluting beads TACE (DEB-TACE), doxorubicin (25 mg/mL) was loaded in 100–300-micrometer LC beads (Boston Scientific, Marlborough, MA, USA). Next, drug-loaded beads (up to 100 mg) were mixed with soluble contrast material (Oxilan, (iodine: 300 mg/mL; Guerbet, Princeton, NJ, USA) and administered until complete delivery or substantial blood-flow reduction into tumor-feeding vessels was achieved. 

### 2.3. Follow-Up

Patients were assessed during a consultation before and 4–6 weeks after each TACE session. At this point, laboratory investigations, a physical examination and contrast-enhanced multiphasic liver MR imaging were performed.

MR imaging Protocol (Supplementary material S1).

### 2.4. Tumor-Response Evaluation

Tumor response was performed using two forms of semiautomatic 3D quantitative software (Philips Research, Medisys, France) [8,9,12]. A radiological reader performed 3D tumor segmentation with the first software on T1-weighted MR images in the arterial phase (20 s after i.v. contrast injection) before and after TACE. The reader semi-automatically used a balloon tool to increase/decrease the tumor segmentation mask in 3D [8]. This semiautomatic tumor-segmentation software demonstrated its reader-independent reproducibility and accuracy in a previous work [13]. The second software provided both the whole-tumor volume (cm^3^) and the enhancing-tumor volume (cm^3^). For this purpose, the naïve unenhanced T1-weighted MR images obtained before contrast material administration were subtracted from the images acquired during the arterial phase to remove any background signal. The 3D segmentation mask that was obtained with the first software was then transposed onto this subtracted MR imaging scan. This segmentation provided the whole-tumor volume. The volume of enhancing tumor was obtained by placing a 1-cubic-centimeter region of interest in the non-tumor liver parenchyma as a reference for normalization to calculate the relative enhancement within the tumor. A color map of the tumor-segmentation mask in 3D was also provided by the software and allowed to visually show enhancing, i.e., viable, tumor regions in red and necrotic non-enhancing tissue in blue [9]. This procedure was performed by two independent readers, neither of whom were involved in the TACEs (mean values were analyzed) [12,14].

Up to 2 targeted lesions were analyzed per patient. Inclusion criteria were: (1) therapy-naïve; (2) visualized without artifacts on MR imaging; and (3) diameter over 1 cm [15]. The qEASL criteria defined the responses of target lesions according to the changes in the enhancing-tumor volume: complete response (CR), disappearance of all enhancing tissue; partial response (PR) and progressive disease (PD), ≥65% decrease and ≥73% increase, respectively, of the sum of target lesions; and stable disease (SD), neither PR nor PD [9]. Patients were defined as responders when they achieved PR or CR and as non-responders when categorized as PD or SD. The patients who showed a response after the first TACE were defined as initial responders. The initial non-responders who showed treatment success with response following the second TACE were defined as secondary responders.

### 2.5. Statistical Analysis

Frequencies and percentages were used for categorical variables and means and ranges for continuous variables. For categorical variables, the Fisher’s exact or Chi-square tests were performed. For continuous variables, parameters were compared using the *t* test. OS corresponded to the time from the first TACE until death (from any cause). Patients who had alternate therapy following repeated TACE, were lost to follow-up, or remained alive were excluded. The Kaplan–Meier method with the log-rank test were used for the survival analysis. Risk factors associated with survival were investigated with the Cox proportional-hazards model. Lab findings/parameters immediately before each TACE were included in the univariate and multivariate analyses. A statistically significant difference was considered with a two-tailed *p*-value < 0.05 (SPSS (SPSS Inc., version 17.0, Chicago, IL, USA).

## 3. Results

The baseline demographics and clinical characteristics of the patients are shown in Table 1. The majority of the patients (82/94, 87.2%) were male and 63 (67%) had hepatitis virus B/C infection. The median age was 62 years (range, 22–87). A median of three TACE sessions (range 1–9) were performed per patient. In total, 58 (61.7%) patients received cTACE; 23 (24.5%) patients were treated with DEB-TACE; and 13 (13.8%) patients received both treatments over time. No unexpected toxicities or TACE-related deaths were observed. A total of 292 procedures were performed and 145 tumors were included in the analysis. At baseline, the mean tumor volume and enhancing-tumor volume were 199.9 ± 379.7 cm^3^ (range, 3.9–2522.1) and 111.0 ± 211.1 cm^3^ (range, 1.1–1549.5), respectively. The mean follow-up time was 25 months (range 2.1–106.2). 

### 3.1. Tumor Response 

**Figure 1:**
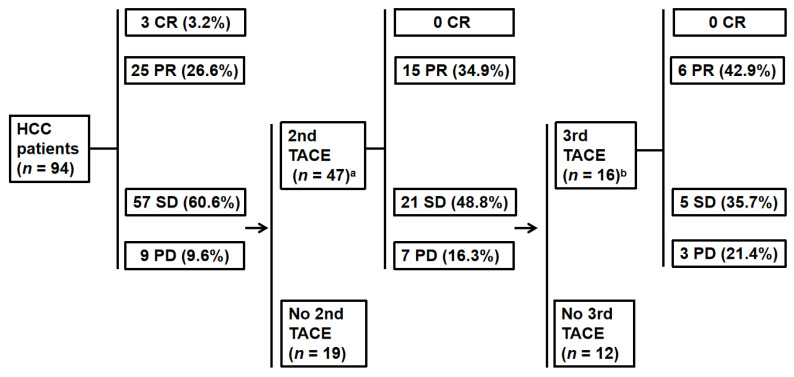
Flow chart summarizing the TACE sessions and tumor responses in the series of HCC patients. ^a^ Four patients without preserved imaging follow-up data after the second TACE; ^b^ two patients without preserved imaging follow-up data after the third TACE.

All 94 patients underwent the first TACE. According to the qEASL criteria, 3 of 94 patients (3.2%) had a CR and 25 of 94 patients (26.6%) had a PR after the first TACE. In total, 66 patients (70.2%) showed non-response, including 57 (60.6%) SD and 9 (9.6%) PD (Figure 1). The mean value of the enhancing-tumor volume in the response group decreased from 95.4 cm^3^ (range, 1.4–1494.4) to 18.5 cm^3^ (range, 0–195.6) (*p* = 0.152).

**Figure 2:**
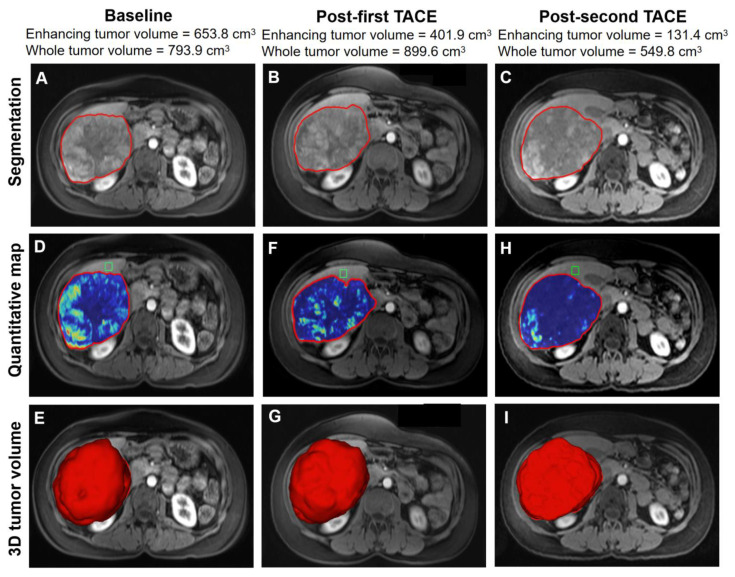
Three-dimensional volumetric semiautomatic evaluation of tumor response after two sessions of TACE therapy in one case. The first row shows the 3D tumor segmentation before treatment, after the first TACE, and after the second TACE treatment, respectively (**A**–**C**). At baseline, the enhancing-tumor volume was 653.8 cm^3^ (**D**) and the whole-tumor volume was 793.9 cm^3^ (**E**). After the first TACE, the patient showed non-response to treatment, with enhancing-tumor volume of 401.9 cm^3^ (**F**), and whole-tumor volume of 899.6 cm^3^ (**G**). However, after the second TACE treatment, the patient responded to TACE. The enhancing-tumor volume decreased significantly to 131.4 cm^3^ (**H**), although the whole-tumor volume remained stable, with a value of 549.8 cm^3^ (**I**). The green box represents the location of the background region of interest.

Of the 66 initial non-responders, 47 (71.2%) received a second TACE within 3 months (median 0.8, range 0.1–3). Six patients received a second TACE after more than 3 months. Thirteen patients refused further TACE treatments. Of the 47 patients who received a second TACE within 3 months, 43 had complete follow-up imaging and were included in the analysis. Fifteen patients (34.9%) who had an initial non-response achieved a response after the second TACE. The mean value of the enhancing-tumor volume decreased from 142.3 cm^3^ (range, 4.2–653.8) to 25.0 cm^3^ (range, 0.8–131.4) (*p* = 0.03). However, the other 28 patients (65.1%) still showed non-response, including 21 SD and 7 PD (Figure 1). Figure 2 shows a case of an initial non-response after the first TACE turned to PR after the second TACE.

Of the 28 non-responders after the second TACE, 16 (57.1%) received a third TACE within 3 months (median 0.7, range 0.1–2.9) (Figure 1). Four patients underwent a third TACE after more than 3 months, and eight patients refused further TACE treatments. A tumor analysis was performed on fourteen patients due to two patients lacking follow-up imaging. Six patients (42.9%) showed a response, whereas eight (57.1%) patients showed no response despite a third treatment. 

### 3.2. Survival Analysis

**Figure 3:**
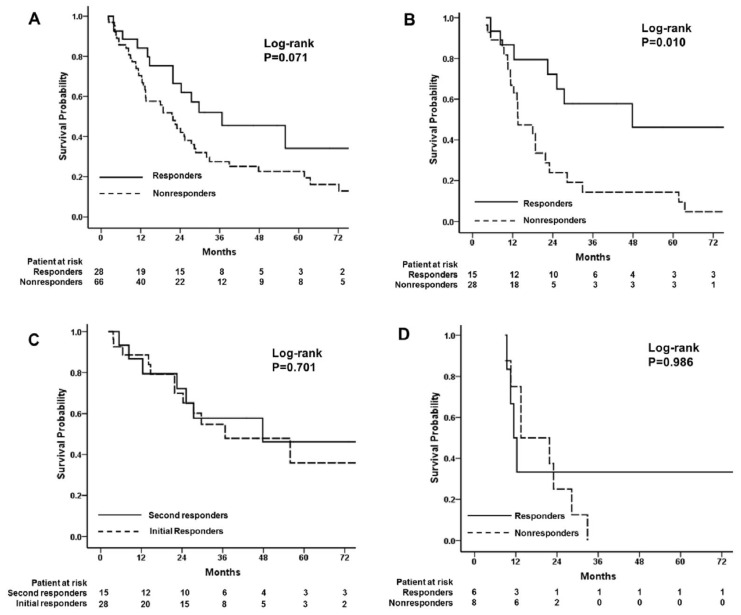
Kaplan–Meier curves showing overall survival (OS) according to the response categories by qEASL. (**A**) Median OS of initial responders was similar to that of non-responders after first TACE treatment. (**B**) Median OS of responders to second TACE session was longer than that of non-responders. (**C**) Median OS was similar between the initial responders and the secondary responders. (**D**) Median OS of responders was similar between responders and non-responders after the third TACE.

At the time the study was conducted, 60 patients (63.8%) had died. The median OS was 24.1 months (95%CI 19.8–28.4). After the first TACE, the OS was similar between the initial-response and non-response groups, although there was a clear positive trend between response and survival (36.7 months (95%CI 9.8–63.6) vs. 21.5 months (95%CI 15.9–27.1), respectively; *p* = 0.071) (Figure 3A). The OS was similar between the initial non-responders who received a second TACE and those who did not (22.5 months (95%CI 15.5–29.5) vs. 21.5 months (95%CI 0.00–48.4), respectively; *p* = 0.831). After the second TACE, the median OS of the responders was significantly longer than that of the non-responders (47.8 months (95%CI 23.8–96.5) vs. 13.6 months (95%CI 8.0–19.2), respectively; *p* = 0.010) (Figure 3B). Furthermore, there was no significant difference in OS between the 28 responders after the first TACE and the 15 responders after the second TACE (36.7 months (95%CI 10.4–63.0) vs. 47.8 months (95%CI 23.8–96.5), respectively; *p* = 0.701) (Figure 3C). The liver function was similar between the initial responders and second responders. There were six patients with Child–Pugh B7 liver function among the 28 initial responders and one patient with Child–Pugh B7 liver function in 15 responders (*p* = 0.391). The OS was similar between the patients who received a third TACE and those who did not (18.0 months (95%CI 11.4–24.6) vs. 13.6 months (95%CI 12.8–14.3), respectively; *p* = 0.616). The difference in OS between the responders and non-responders after the third TACE was not significant (11.4 months (95%CI 9.2–13.6) vs. 13.5 months (95%CI 3.2–23.8), respectively; *p* = 0.986) (Figure 3D).

### 3.3. Univariate and Multivariate Analysis

At the time of the first TACE, the patients with Child–Pugh class B had a significantly worse survival compared to the Child–Pugh class A patients (hazard ratio (HR) of 2.7 (95%CI 1.5–4.7; *p* = 0.01) and 2.5 (95%CI 1.1–5.7; *p* = 0.036) in the univariate and multivariate analyses, respectively) (Table 2). Ascites and qEASL response reached *p* < 0.10 in the univariate analysis (*p* = 0.02 and *p* = 0.075, respectively), but failed to show significance in the multivariate analysis (Table 2). 

After the second TACE, the univariate analysis showed that Child–Pugh class B (HR = 3.4 (95%CI 1.5–7.6)), ascites (HR = 2.5 (95%CI 1.1–5.6)) and non-response (HR = 2.8 (95%CI 1.2–6.4)) were associated with worse OS (Table 2). However, in the multivariate analysis, non-response was the only statistically significant predictor of decreased survival (HR = 2.8 (95%CI 1.2–6.4); *p* = 0.017), with Child–Pugh class B showing a clear trend toward worse prognosis (HR = 3.37 (95%CI 1.5–7.6); *p* = 0.062) (Table 2).

## 4. Discussion

The main finding of our study is that we quantitatively demonstrated that tumor response and survival benefits in intermediate-stage HCC patients cannot always be achieved after one session of TACE, and a second TACE is beneficial for initial non-responders. 

It remains unclear how many TACE sessions should be performed. Currently, there are no universally accepted guidelines or randomized controlled studies that clarify this issue. Moreover, although TACE essentially targets cancer tissue, unavoidably, part of the treatment may injure the liver parenchyma, and there is concern that multiple TACEs may unfavorably affect liver function and vasculature, and prevent additional therapy [16]. Therefore, it is crucial to investigate whether additional rounds of TACE are useful for patients in order to ensure that they are not prematurely switched to another therapy.

Terzi et al. compared survival and response rates to initial and repeated cTACE [3]. The survival between the responders and non-responders at each TACE session was not significantly different. However, the authors included heterogeneous HCC patients with BCLC stage A to D. Several factors, such as liver function, tumor burden, and ECOG may affect the prognosis of HCC patients. Thus, the results reporting similar OS between responders and non-responders after each TACE may not be reliable. Another potential explanation for the negative results may lie in the study design: the time variable for repeated TACE was not controlled and may thus have caused bias in the analysis of the survival outcomes. In our study, we controlled the time variable by only including patients who received TACE treatment within 3 months of their initial non-response, which helped decrease the bias. 

Georgiades et al. evaluated the response to TACE after initial non-response through mRECIST and EASL [5]. The results showed that the patients who did not respond to the first TACE did not necessarily fail to respond to the second attempt. More recently, Chen et al. also investigated the optimal number of TACEs using mRECIST in a large cohort of intermediate-stage HCC patients [6]. The response rate after the first TACE was 36.7%, and increased to 48.4% after the second session in the non-responders who had stable disease after the initial treatment. When stable disease was still observed after the second TACE, the response rate after the third TACE increased to 48.5%. However, the response rate dramatically decreased to <10% in the non-responders to the third, fourth, and fifth TACE. Moreover, the patients with progressive disease who did not respond to their next TACE session showed a response rate of <5%. The survival was significantly higher in the responders to first, second, and third TACE when compared to the non-responders; however, this was not the case for the responders to the fourth session [6].

Unlike the previous studies, which utilized conventional 1D/2D tumor-response criteria (mRECIST [3,6]; mRECIST and EASL [5]), we used the 3D quantitative tumor-response assessment method (qEASL), which has been proven to be more accurate in the tumor analysis and survival prediction following TACE [9,10,11,17,18]. Furthermore, the qEASL proved to be reproducible between radiologic readers [13]. Most importantly, a radiologic–pathologic analysis demonstrated the precision of qEASL, which accurately depicted viable (i.e., enhancing areas) and necrotic (i.e., non-enhancing lesion regions) tumor tissue following TACE with strong correlation on histopathology [12]. In our study, we evaluated the initial non-response to the previous TACE and compared the survival between the response and non-response groups after further TACE treatment in patients with intermediate-stage HCC, preserved liver function, and ECOG 0. After the first TACE, the OS was similar between the initial-response and non-response groups. However, 35% of the patients who were initially non-responders became responders after the second TACE and had significantly longer survival than the non-responders. The multivariate analysis showed that the tumor response was also an independent predictor of OS after the second TACE. Similarly to Chen et al. [6], we found that there was no significant difference in OS between the responders after the first and the second TACE, highlighting the survival benefit of repeated TACE.

Some experts suggest that non-response to three sessions of TACE within 6 months should be defined as TACE failure [19]. In our study, we demonstrated that the non-responders to the second TACE had a poor median OS, of 13.6 months, which is similar to the reported survival data in patients with advanced-stage HCC [20,21]. Moreover, our results showed no survival difference between the responders and non-responders to the third TACE. This contradicts the findings of Chen et al., who showed that the survival of patient responders to the third TACE was comparable to—albeit shorter than—that of responders to the initial TACE [6]. Although we cannot draw a definite conclusion because of the small number of patients who underwent a third TACE in our cohort, our findings using quantitative tumor analysis suggest that at least two TACE sessions should be performed in intermediate-stage HCC. Further studies with a larger patient cohort undergoing at least three TACEs are needed.

There were several limitations to our study. This first was the retrospective design. However, the selection bias was decreased as we used a prospectively collected database. Second, we focused on qEASL, and we did not compare it with other tumor-response criteria, such as mRECIST. Further studies should be conducted to compare the outcomes of different tumor-response assessment methods after repeated TACE. Moreover, another topic of research worth mentioning is the investigation of existing scores with tumor-response criteria in the setting of repeat TACE [22]. Third, our cohort was relatively small, particularly after the third TACE. This might have produced an underestimation of the statistical difference between the response and non-response groups. Further studies with a larger cohort of patients undergoing ≥3 TACEs are needed.

In conclusion, we demonstrated that patients who do not respond to the first TACE treatment can benefit from a second TACE in terms of survival, as determined by 3D tumor-response assessment criteria (qEASL). Our data quantitatively confirm that at least two TACEs should be performed before therapy is abandoned. Further investigation with a larger sample size is needed to determine whether non-responders to a second TACE could benefit from a third TACE treatment.

## Figures and Tables

**Table 1:** Patient baseline demographics and clinical characteristics (*n* = 94).

Variable	No. (%)
Age/years, median (range)	62 (22–87)
Male	82 (87.2%)
Etiology	
Hepatitis B/C infection	63 (67.0%)
Other	31 (33.0%)
Cirrhosis	
Yes	69 (73.4%)
No/unknown	25 (26.6%)
Child–Pugh class	
A (5–6)	71 (75.5%)
B (7)	23 (24.5%)
Ascites	
No	76 (80.9%)
Yes	18 (19.1%)
No. of HCC nodules	
1–2	41 (43.6%)
≥3	53 (56.4%)
AFP *	
<400 ng/dL	68 (72%)
≥400 ng/dL	25 (27%)
Baseline laboratory values, mean (range)	
International normalized ratio	1.1 (0.9–1.8)
Albumin, g/dL	3.8 (2.3–4.8)
Total bilirubin, mg/dL	1.1 (0.2–4.6)
Post-TACE therapies	
Liver transplant	13 (13.8%)
Liver resection	2 (2.1%)
Sorafenib	68 (72.3%)

HCC, hepatocellular carcinoma; AFP, α-fetoprotein; * the AFP value was not available for one patient.

**Table 2:** Univariate and multivariate analyses considering survival at each TACE cycle.

Variable	First TACE						Second TACE					
	Univariate Analysis			Multivariate Analysis			Univariate Analysis			Multivariate Analysis		
	HR	95% CI	*p*	HR	95% CI	*p*	HR	95% CI	*p*	HR	95% CI	*p*
Age	1.012	0.991–1.034	0.250				1.016	0.986–1.047	0.293			
Gender (male/female)	1.081	0.489–2.387	0.848				0.899	0.343–2.360	0.829			
Etiology (hepatitis infection/other)	1.294	0.755–2.218	0.348				0.92	0.444–1.907	0.822			
AFP (≥400/<400)	1.473	0.849–2.556	0.168				1.098	0.533–2.261	0.8			
Child–Pugh (B/A)	2.658	1.498–4.715	0.001	2.450	1.060–5.664	0.036	3.370	1.499–7.576	0.003	3.472	0.942–12.803	0.062
Ascites (Yes/no)	1.707	1.088–2.680	0.02	0.985	0.493–1.967	0.965	2.495	1.112–5.597	0.027	0.923	0.256–3.325	0.902
Tumor number (≥3/1–2)	1.408	0.826–2.401	0.208				1.227	0.525–2.870	0.637			
ECOG (1/0) *	-	-	-	-	-	-	0.747	0.300–1.859	0.53			
qEASL (non-response vs. response)	1.724	0.946–3.141	0.075	1.502	0.809–2.789	0.197	2.817	1.238–6.411	0.014	2.756	1.196–6.352	0.017

HCC, hepatocellular carcinoma; HR, hazard ratio; CI, confidence interval; PVTT, portal vein tumor thrombosis; ECOG, Eastern Cooperative Oncology Group; TACE, transarterial chemoembolization; qEASL, quantitative European Association for the Study of the Liver. * ECOG does not apply to first TACE, since all patients were ECOG 0 at baseline.

## Data Availability

The raw data supporting the conclusions of this article will be made available by the authors, without undue reservation.

## Supplementary Materials

The following supporting information can be downloaded at: https://www.mdpi.com/article/10.3390/cancers14153615/s1, Supplementary material S1: MRI Protocol.

## Abbreviations

HCC, hepatocellular carcinoma; TACE, transarterial chemoembolization; BCLC, Barcelona Clinic Liver Cancer; RECIST, Response Evaluation Criteria in Solid Tumors; mRECIST, modified RECIST; EASL, European Association for the Study of the Liver; qEASL, quantitative EASL; ECOG, Eastern Cooperative Oncology Group; CR, complete response; PR, partial response; SD, stable disease; PD, progression disease; DEB, drug-eluting bead; OS, overall survival.
