# Supplementary material for: Three-Dimensional Quantitative Tumor Response and Survival Analysis of Hepatocellular Carcinoma Patients Who Failed Initial Transarterial Chemoembolization: Repeat or Switch Treatment?
Source: Cancers (Basel). 2022 Jul 25;14(15):3615. doi: 10.3390/cancers14153615 (PMC9329887; doi:10.3390/cancers14153615)
Supplement: Supplementary file 1 [file cancers-14-03615-s001.zip › cancers-1780499 - supplementary.pdf]

cancers

MDPI

Supplementary Material S1: MRI Protocol

MR imaging was performed at baseline and 3–6 weeks after each TACE by using a 1.5-T MRI unit (CV/I; GE Medical Systems, Milwaukee, WI, USA) and a phased-array torso coil for signal reception. The standardized liver protocol included axial breath-hold unenhanced and contrast-enhanced (0.1 mmol per kilogram of body weight of intravenous gadodiamide [Omniscan, GE Healthcare, Princeton, NJ, USA]) T1-weighted three-dimensional fat-suppressed spoiled gradient-recalled echo images (5.1/1.2; field of view, 320–400 mm^2; matrix size, 192 × 160; section thickness, 4–6 mm; receiver bandwidth, 64 kHz; flip angle, 15°) in the arterial, portal venous and equilibrium phases (20, 60 and 180 seconds after intravenous contrast material injection, respectively).
